# Long-time evolution and highly dynamic satellite DNA in leptodactylid and hylodid frogs

**DOI:** 10.1186/s12863-014-0111-x

**Published:** 2014-10-15

**Authors:** Stenio Eder Vittorazzi, Luciana Bolsoni Lourenço, Shirlei Maria Recco-Pimentel

**Affiliations:** Departamento de Biologia Estrutural e Funcional, Instituto de Biologia, Universidade Estadual de Campinas, 13083-863 Campinas, São Paulo, Brazil

**Keywords:** Satellite DNA, Chromosomes, *Physalaemus*, Amphibia

## Abstract

**Background:**

Satellite DNA sequences are the most abundant components of heterochromatin and are repeated in tandem hundreds to thousands of times in the genome. However, the number of repeats of a specific satellite family can vary even between the genomes of related species or populations. The PcP190 satellite DNA family was identified in the genome of the leptodactylid frog *Physalaemus cuvieri,* which showed to be derived most likely from the 5S rDNA in an ancestral species. In this study, we investigate the presence of the PcP190 satellite DNA in several *P. cuvieri* populations and in four closely related species at the chromosomal and molecular level. Furthermore, we investigate the occurrence of this satellite DNA in the genomes of *P. marmoratus* as well as in representative species of the leptodactylid genus *Leptodactylus* (*L. latrans*) and the hylodid family (*Crossodactylus gaudichaudii*), all with the aim of investigating if the PcP190 satellite DNA presents or not a restricted distribution.

**Results:**

The PcP190 satellite DNA was detected in all the analyzed species. Some of them exhibited particular sequence differences, allowing the identification of species-specific groups of sequences, but in other species, the sequences were more conserved. However, in a general analysis, conserved and variable domains have been recognized within the PcP190 monomer. The chromosomal analysis performed on *P. cuvieri* populations and closely related species revealed high variability of the satellite DNA amount and its chromosomal location, which has always been coincident with regions of centromeric/pericentromeric heterochromatin.

**Conclusion:**

The PcP190 satellite DNA was found in representatives of two families, Leptodactylidae and Hylodidae, indicating that these sequences are widely distributed and conserved in these frogs. There is a pattern of non-random variation within the repeating units, indicating interplay between stochastic events and selective pressure along the PcP190 sequences. Karyotypic differences involving the PcP190 satellite DNA prove to be highly dynamic on the chromosomes of the *Physalaemus* and its differential accumulation has contributed to the differentiation process of the Z and W sex chromosomes in *P. ephippifer*.

## Background

Repetitive DNA sequences have been detected in the genomes of almost all eukaryotes, and a wide variety of characteristics distinguish them into different classes, including satellite DNA, transposable elements and some multigene families. The satellite DNA sequences, which are repeated in tandem hundreds to thousands of times in the genome, are found predominantly in the centromeric, pericentromeric and telomeric regions and are main components of constitutive heterochromatin [1,2].

Families of satellite DNA are distinguished by nucleotide composition, sequence complexity, the size of the repeating unit and the number of copies, and they share the ability to form arrays of long sequences arranged in tandem to form heterochromatic regions [3]. Importantly, distinct families of satellite DNA sequences can also differ in evolutionary rates. While some satellite DNA families are species-specific [4,5], others are more conserved, and similar sequences may be recognized in closely related species [6-9].

The dynamic evolutionary processes that affect satellite DNA may also result in changes in its chromosomal location and distribution. In some cases, these changes can correlate with chromosomal evolution and possibly influence species evolution [10-14]. The same type of satellite DNA can be found in both homologous and non-homologous chromosomal regions [5,9,15], and all the satellite DNA clusters in a genome can evolve in concert, which leads to the homogenization of the satellite DNA families in the genome [2,16-18].

The principle of concerted evolution of repetitive sequences is based on different mechanisms of non-reciprocal transfer occurring within or between chromosomes, such as unequal crossover, gene conversion and transposition [17,18]. In addition, the model of concerted evolution also takes into account the fixation of specific satellite DNA sequences in populations/species that is driven by sexual reproduction. Also based on this model, the members of a satellite DNA family in a given species are expected to be more similar to each other than with those of related species [17,18].

Related species may share a library of satellite DNA sequences, and in each species the copy number of a set of sequences may be amplified or not. This is the basis of the “library model”, which was originally proposed by Salser et al. [19] and Fry and Salser [20] and provides an explanation for the occurrence of species-specific profiles, as has been corroborated by several studies ([21-24], reviewed in [12]). The extent of change in copy number can vary even among closely related species and this phenomenon may be accompanied by changes in nucleotide sequence [3,12,24].

In the frog *Physalaemus cuvieri*, a family of satellite DNA named PcP190 was characterized and shown to be derived most likely from the 5S rDNA in an ancestral species. The clusters of satellite DNA are present in the centromeric/pericentromeric regions of at least five chromosome pairs in the population from Palmeiras, Bahia (BA) State, Brazil. However, the occurrence and chromosomal distribution of the PcP190 satellite DNA family in other species of *Physalaemus* is not known [25].

The genus *Physalaemus* belongs to the subfamily Leiuperinae of Leptodactylidae and is currently composed of 46 species [26], nine of which are found in South America and belong to the *P. cuvieri* group: *P. albonotatus*, *P. centralis*, *P. cicada*, *P. cuqui*, *P. cuvieri*, *P. ephippifer*, *P. erikae*, *P. fischeri* and *P. kroyeri* [26,27]. *Physalaemus albifrons* was previously assigned to the *P. cuvieri* group [28], but based on phenetic analysis it was reallocated to form the *P. albifrons* group along with *P. biligonigerus*, *P. marmoratus*, *P. santafecinus* and *P. riograndensis* [27]. None synapomorphy, however, was clearly recognized for this species group, and Vittorazzi et al. [29] proposed to maintain *P. albifrons* in *P. cuvieri* group based on chromosomal data.

Except for *P. erikae* and *P. fischeri*, all the species of the *P. cuvieri* group have already been studied cytogenetically, and they have a conservative karyotype with a diploid number of 22 chromosomes [29-35]. Despite this conserved karyotype, differences at the chromosomal positions of the nucleolus organizing regions (NORs) were detected among these species and even among different populations of *P. cuvieri* [29,34]. In addition, the karyotype of specimens of *P. cuvieri* from Porto Nacional, Tocantins State (TO), Brazil, could be distinguished from that found in specimens from other localities by the C-banding pattern [34]. Therefore, the genus *Physalaemus* is an interesting group for cytogenetic studies and for evaluating the usefulness of new chromosomal markers.

In this study, we report on a preliminary investigation of the extent of occurrence of the PcP190 satellite DNA in anurans, with emphasis on the characterization of PcP190 chromosomal sites and sequences in the genus *Physalaemus*. We analyzed five populations of *P. cuvieri* together with samples from four other species considered by Nascimento et al. [27] and Vittorazzi et al. [29] to be closely related to *P. cuvieri* (*P. albifrons*, *P. albonotatus*, *P. centralis*, and *P. ephippifer*). To investigate the occurrence of PcP190 satellite DNA in less closely related species, we also verified the occurrence of these sequences in *P. marmoratus*, as well as the leptodactyline species *Leptodactylus latrans* and the hylodid *Crossodactylus gaudichaudii*.

## Methods

### Specimens

All the individuals belonging to eight species included in our analyses were deposited in the Museum of Zoology “Professor Adão José Cardoso” of the Universidade Estadual de Campinas (ZUEC). We analyzed ten individuals of *P. cuvieri* from five Brazilian localities [Uberlândia, Minas Gerais State (MG; ZUEC 13367); Passo Fundo, Rio Grande do Sul State (RS; ZUEC 14650); Porto Nacional, Tocantins State (TO; ZUEC 14691, ZUEC 14692, ZUEC 14694, ZUEC 14695, ZUEC 14699 and ZUEC 14702); Araruna, Paraíba State (PB; ZUEC 17899); and Três Lagoas, Mato Grosso do Sul State (MS; ZUEC 17548)], two individuals of *P. centralis* [Palestina, São Paulo State (SP; ZUEC 13689 and ZUEC 13692)], two individuals of *P. albonotatus* [Lambari D’Oeste, Mato Grosso State (MT; ZUEC 16418 and ZUEC 16419)]*,* five individuals of *P. ephippifer* [Belém, Pará State (PA; ZUEC 17729♀, ZUEC 17737♀, ZUEC 13739♀, ZUEC 13741♀ and ZUEC 13734♂)] and two individuals of *P. albifrons* [one from Barreirinhas, Maranhão State (MA; ZUEC 17925) for the cytogenetic analysis and one from Alagoinhas, Bahia State (BA; ZUEC 17902) for dot-blotting and sequence analyses].

The molecular analyses also included samples of the leptodactylid species *P. marmoratus* [Itirapina, São Paulo state (SP; ZUEC 17515) and *L. latrans* [Bertioga, São Paulo state (SP; ZUEC 12875)], as well as the hylodid *C. gaudichaudii* [Rio de Janeiro, Rio de Janeiro State (RJ; ZUEC 17570)].

**Figure 1:**
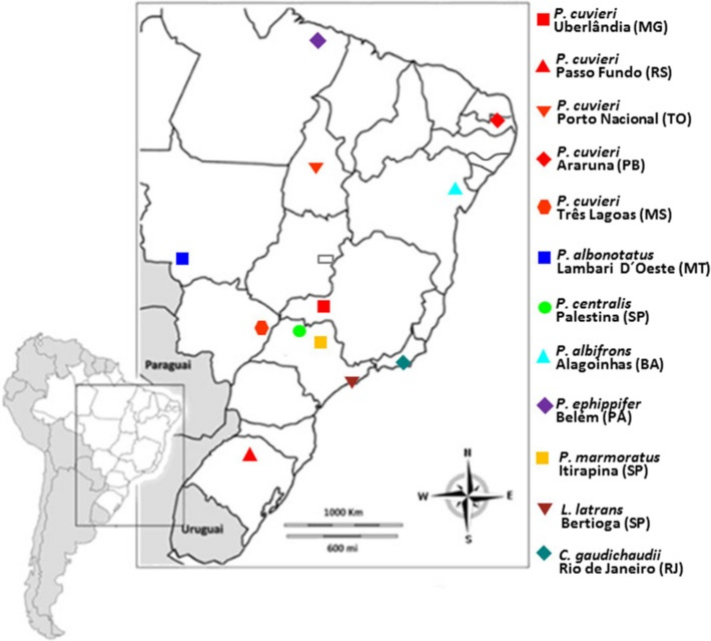
**The collecting localities of the specimens analyzed in the present study.** Brazilian states: BA = Bahia; MG = Minas Gerais; MS = Mato Grosso do Sul; MT = Mato Grosso; PA = Pará; PB = Paraíba; RJ = Rio de Janeiro; RS = Rio Grande do Sul; SP = São Paulo; TO = Tocantins.

The collection localities of all the specimens analyzed are shown in Figure 1. The animals were collected with permission of the Instituto Brasileiro do Meio Ambiente e dos Recursos Naturais Renováveis (IBAMA/SISBIO – Process number 10678–2, 20336–1 and 33133–1). For the subsequent techniques, all samples were extracted from euthanized specimens using anesthetic application to the skin (5% Lidocaine) to minimize animal suffering, according to recommendations of the Herpetological Animal Care and Use Committee (HACC) of the American Society of Ichthyologists and Herpetologists (available in http//www.asih.org), and approved by SISBIO/Institute Chico Mendes de Conservação da Biodiversidade as a condition for the concession license.

### Extraction of genomic DNA

The genomic DNA was extracted from liver or muscle samples maintained at −80°C. Tissue fragments were lysed in TNES (50 mM Tris pH 7.5, 400 mM NaCl, 20 mM EDTA and 0.5% SDS) supplemented with proteinase K (100 μg/mL) at 56°C for approximately 3 hours. After lysis, the samples were treated with RNAse (50 μg/mL) and NaCl was added to a final concentration of ~1.7 M. The DNA was precipitated in isopropyl alcohol, washed in ethanol (70%) and rehydrated in TE (10 mM Tris–HCl, 1 mM EDTA, pH 8). For quality control and to quantify the genomic DNA, the samples were subjected to electrophoresis in a 0.8% agarose gel and spectrophotometry.

### Isolation, cloning and sequencing of the PcP190 satellite DNA

To isolate the satellite DNA of each species, DNA samples were subjected to PCR using specific primers: P190F (AGA CTG GCT GGG AAT CCC AG) and P190R (AGC TGC TGC GAT CTG ACA AGG) [20]. Subsequently, the DNA fragments were inserted into the pGEM-T Easy Vector (Promega, Madison, Wisconsin, USA). The recombinant vectors were transformed into JM109 *E. coli* with the TransformAid™ Bacterial Transformation Kit (Fermentas, Burlington, Ontario, Canada) according to the manufacturer’s protocol.

The bacterial suspensions were plated on solid LB containing ampicillin (100 μg/mL), X-gal (2% in dimethylformamide) and IPTG (50 mM) and were incubated overnight at 37°C. The white colonies were cultivated at 37°C for approximately 16 hours on a new plate containing solid LB medium supplemented with ampicillin. A sample of each colony was subjected to PCR using the T7 and SP6 primers to confirm the presence of the insert. The rest of each colony was suspended in liquid LB medium supplemented with ampicillin (100 μg/mL). After two hours, the liquid cultures were used for plasmid DNA extraction following the method described by Sambrook et al. [36]. A sample of each liquid culture was stored at −80°C with glycerol (30% v/v).

Samples of the PCR amplified fragments were subjected to sequencing reactions with the BigDye Terminator kit (Applied Biosystems, Foster City, California, USA) and purified by DNA precipitation. The samples were dried, suspended in loading dye [Blue-Dextran-EDTA/Formamide (1:5)], denatured for 3 minutes at 94°C and applied to an automatic sequencer (ABI 3730XL DNA Analyzer*,* Applied Biosystems, Foster City, California, USA).

### Sequences analysis

The nucleotide sequences were edited using the BioEdit program v. 7.0.9 [37] and aligned using Clustal W [38]. The sequences were compared with each other and with those from *P. cuvieri* from Palmeiras, Bahia State, Brazil [25] available in the GenBank, and the averages of pairwise comparisons were presented in percentage. Furthermore, the sequences were compared using the Maximum Likelihood criterion based on the Kimura 2-parameter model [39] using MEGA v5 [40]. Initial trees for the heuristic search were obtained automatically. The inferred arrangements were evaluated by bootstrap analysis using 1000 replicates.

### Dot-blot analysis

To compare the abundance of the PcP190 satellite in the genomes of *P. albifrons*, *P. albonotatus*, *P. centralis*, *P. ephippifer* and *P. cuvieri*, 100 ng, 250 ng and 500 ng of genomic DNA from each species were blotted onto a positive nylon membrane. The membrane was treated with NaOH (1 M) and baked at 80°C for 2 hours to denature the DNA. The membrane was then neutralized in NaCl (1.5 M), Tris–HCl (0.5 M) pH 7.5 for 30 minutes. The PcP190 satellite DNA isolated from *P. cuvieri* [19] was labeled with digoxigenin by PCR and used as a probe to hybridize to the membrane overnight at 65°C. The membrane washing was made first at room temperature in SSC (2X) and SDS (0.1%) by 5 minutes and then at 65°C in SSC (0.1X) and SDS (0.1%) by 15 minutes. The probe was detected using the DIG Nucleic Acid Detection kit (Roche, Penzberg, Bavaria, Germany) according to the manufacturer’s instructions. To estimate the percentage in each genome, PCR sample of PcP190 were blotted onto a positive nylon membrane in the same concentrations above mentioned, thus considering that each one of them has 100% of the hybridization signal, the intensity of them were measured by densitometry using the ImageJ program [41] and proportions were established.

### Chromosome preparations, fluorescence *in situ* hybridization (FISH) and the Ag-NOR method

Mitotic metaphases were obtained from cell suspensions of intestinal epithelium from the individuals previously treated with colchicine ([42,43], with modifications). To obtain the FISH probes, cloned fragments were PCR amplified in the presence of digoxigenin-dUTP (Roche, Penzberg, Bavaria, Germany). The probes were mixed with salmon DNA (1 ng/μL of probe) and precipitated with ethanol. All the resulting DNAs were dissolved in a hybridization buffer at pH 7 that was composed of deionized formamide (50%), 2x SSC, phosphate buffer (40 mM), Denhardt’s solution, SDS (1%) and dextran sulfate (10%). The *in situ* hybridization technique followed Viegas-Péquignot [44] with modifications for the detection of digoxigenin-labeled probes with anti-DIG-Rhodamine (Roche, Penzberg, Bavaria, Germany).

Images of the hybridized metaphase chromosomes were captured with an Olympus BX-60 (Tokyo, Japan) microscope and edited with the Image-Pro Plus program (Media Cybernetics, Rockville, Maryland, USA). The chromosome pairing of all species followed Vittorazzi et al. [29], Quinderé et al. [34] and Nascimento et al. [35]. The nucleolus organizer regions (NOR) in the karyotype of *P. ephippifer* were detected by the Ag-NOR method following Howell and Black [45].

## Results

### PcP190 Satellite DNA sequences

For all the analyzed species, the PCR using the P190F and P190R primers amplified multiple DNA bands, which is typical for satellite DNA. The majority (62%) of the cloned sequences included partial PcP190 monomers, but inserts with dimers (23%), trimers (12%) and tetramers (2%) were also obtained. Although all the cloned fragments were sequenced, we used only the complete PcP190 monomers in the comparative analyses; thus, partial sequences resulting from the amplification of a single PcP190 monomer were not included. However, it is worth noting that these partial units showed no relevant difference in their nucleotide composition in relation to the complete sequences.

**Figure 2:**
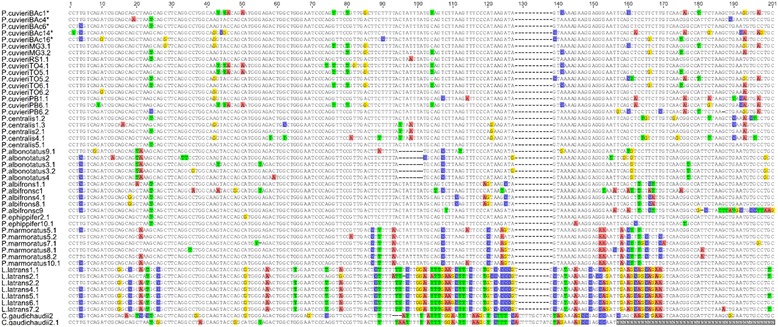
**Alignment of the 48 PcP190 satellite DNA monomers.** The sequences are labeled with the species names, followed by the clone number. In *P. cuvieri*, the acronyms (two capital letters) correspond to the respective Brazilian state, as in Figure 1. Colored sites differ from the nucleotide recorded in the majority of samples, and gaps indicate indels. Sequences extracted from GenBank (JF281121, JF281117, JF281124 and JF281119) are indicated by an asterisk. The accession numbers of the other samples are KM361673–KM361706 and KM361718–KM361726. This figure was created in Geneious 7.1 (Biomatters).

A total of 43 full monomers were obtained: 11 from the different populations of *P. cuvieri,* 5 from *P. centralis*, 5 from *P. albonotatus*, 5 from *P. albifrons*, 2 from *P. ephippifer,* 6 from *P. marmoratus*, 7 from *L. latrans* and 2 from *C. gaudichaudii* (exceptionally, one incomplete sequence from this last species was included due to the low number of complete sequences isolated from this species) (Figure 2). When compared with the other sequences available in GenBank, the sequences isolated here were similar to the PcP190 sequences previously isolated from *P. cuvieri* (Palmeiras, Bahia State, in Brazil) [25] (Figure 2), suggesting that all of them belong to the same satellite DNA family. Some differences were noted among the isolated sequences, but these differences were not enough to mischaracterize the family of the PcP190 satellite DNA (Figure 2). The main feature of this satellite DNA family is the presence of nucleotide substitutions, with a transition frequency of 11.29% and a transversion frequency of 6.86%. Most of the sequences are 190 bp long and have a 52% A/T content.

All the sequences of the *Physalaemus* species are very similar and most have 189–190 bps. The PcP190 monomers of *P. albonotatus* are 183 bps long, differing in size from the sequences obtained from the other *Physalaemus* species by the absence of seven consecutive base pairs (Figure 2). The PcP190 sequences isolated in *P. cuvieri* and the closely-related species *P. albifrons*, *P. albonotatus*, *P. centralis*, and *P. ephippifer* are more similar to one other (interspecific pairwise similarity: 90–95%) than to those obtained from *P. marmoratus*, with pairwise similarity ranging between 87% and 89.6% (Figure 2).

The PcP190 sequences of *L. latrans* and *C. gaudichaudii* varied considerably in the region between positions 94 and 169 (Figure 2), both in comparison with each other and also when compared to the *Physalaemus* sequences. However, this variable region is flanked by sequences that are very similar to those found in *Physalaemus* (Figure 2).

**Figure 3:**
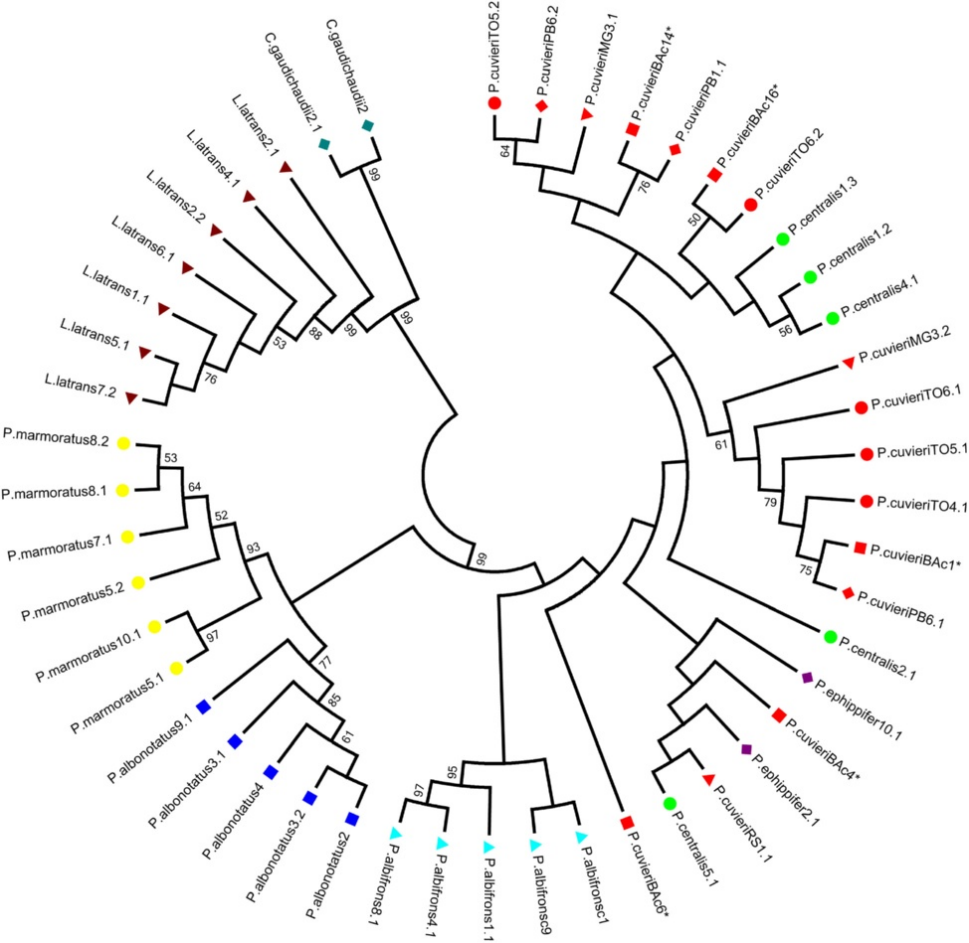
**Maximum likelihood analysis of the PcP190 satellite DNA in** ***Physalaemus*** **species.** Dendrogram of the evolutionary relationships among the PcP190 satellite DNA sequences recorded in the six *Physalaemus* species, *L. latrans*, and *C. gaudichaudii*. Species are differentiated by symbols and colors. The *P. cuvieri* populations are differentiated by red symbols. *Sequences from GenBank – JF281121, JF281117, JF281109, JF281124 and JF281119 [25]. The numbers are bootstrap values (those lower than 50 were omitted).

The maximum likelihood analysis of the PcP190 sequences identified species-specific patterns in the sequences from *L. latrans*, *C. gaudichaudii, P. albonotatus, P. albifrons*, and *P. marmoratus* (Figure 3). The distinctive pattern observed in *P. albonotatus* was related primarily to the 7-bp indel mentioned above. By contrast, the sequences isolated from *P. cuvieri*, *P. centralis*, and *P. ephippifer* were not species-specific and clustered together in the same group (Figure 3).

The amounts of the PcP190 sequences inferred by the dot-blot experiments varied among the genomes of *P. cuvieri* and its closely related species analyzed here. The lowest abundance value (1.6% of the genome) was estimated for *P. albonotatus,* and the largest abundance (6.5% of the genome) was observed in *P. cuvieri* from Três Lagoas (MS) (Table 1).

**Table 1:** **Percentage estimation of dot-blot hybridization of PcP190 satellite DNA**

**Species**	**Localities**	**PcP190 estimation (%) in the genome**
*Physalaemus cuvieri*	Uberlândia (MG)	5,3
*Physalaemus cuvieri*	Passo Fundo (RS)	1,7
*Physalaemus cuvieri*	Porto Nacional (TO)	5,9
*Physalaemus cuvieri*	Araruna (PB)	6,1
*Physalaemus cuvieri*	Três Lagoas (MS)	6,5
*Physalaemus albifrons*	Alagoinhas (BA)	2,4
*Physalaemus albonotatus*	Lambari D’Oeste (MT)	1,6
*Physalaemus centralis*	Palestina (SP)	2,7
*Physalaemus ephippifer*	Belém (PA)	2,2

### PcP190 mapping in the karyotypes of *P. cuvieri* and closely related species

In the karyotypes of *P. cuvieri, P. albifrons*, *P. albonotatus*, *P. centralis* and *P. ephippifer*, the fluorescence *in situ* hybridization detected some chromosomal regions harboring the PcP190 satellite DNA that were regions of centromeric and pericentromeric constitutive heterochromatin.

**Figure 4:**
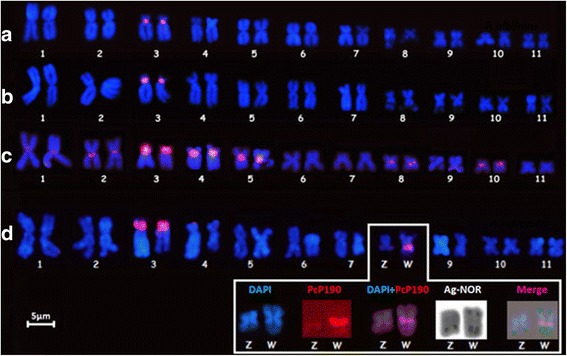
**Karyotypes of** ***Physalaemus*** **species hybridized with the PcP190 satellite DNA.** Karyotypes of **(a)** *P. albifrons*, **(b)** *P. albonotatus*, **(c)** *P. centralis*, and **(d)** *P. ephippifer*. In *P. ephippifer,* the inset shows the stronger signal from the W chromosome and the weaker one from the Z chromosome, as well as the distinction between the PcP190 and NOR regions detected by the Ag-NOR method (the signal was artificially colored green in the combined image shown in the inset).

In *P. albifrons* and *P. albonotatus*, the PcP190 probe hybridized to the centromeric/pericentromeric regions of chromosome pair 3 (Figure 4a, b). In the *P. centralis* karyotype, PcP190 hybridized to the centromeric regions of chromosome pairs 1 through 5, 8 and 10 (Figure 4c). In four females of *P. ephippifer*, in addition to the centromeric/pericentromeric regions of chromosome pair 3, the PcP190 probe also detected the pericentromeric C-band in the long arm of the Z and W sex chromosomes. The signal on chromosome W was notably stronger than the signal on chromosome Z in 10 of the analyzed metaphases and was undetectable on chromosome Z in four of the analyzed metaphases (Figure 4d).

**Figure 5:**
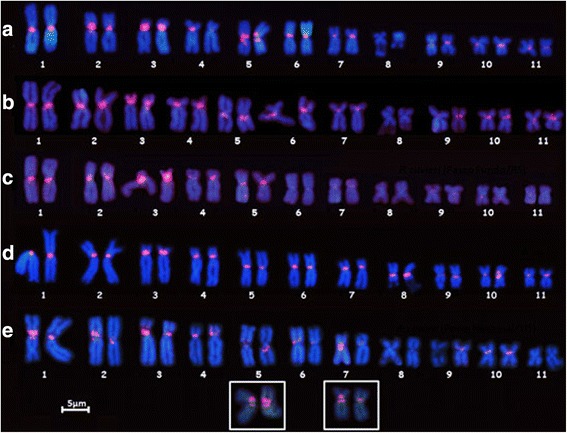
**Karyotypes of the** ***P. cuvieri*** **specimens from different localities hybridized with the PcP190 satellite DNA.** *Physalaemus cuvieri* populations from **(a)** Uberlândia, Minas Gerais (MG), **(b)** Três Lagoas, Mato Grosso do Sul (MS), **(c)** Passo Fundo, Rio Grande do Sul (RS), **(d)**, Araruna, Paraíba (PB), and **(e)** Porto Nacional, Tocantins (TO). The insets in **(e)** present additional details on the signals in pairs 5 and 7.

The karyotypes of the individuals of *P. cuvieri* from distinct populations exhibited conspicuous differences in the hybridization pattern of the PcP190 satellite DNA. In the karyotypes of the specimens from Uberlândia (MG) and Três Lagoas (MS), the PcP190 probe hybridized to the centromeric regions of all the chromosomes, except those of chromosome pair 8 (Figure 5a and b, respectively). In the karyotypes of individuals from Passo Fundo (RS), the PcP190 probe hybridized to the centromeres of chromosome pairs 1 through 5 and weakly to some of the smaller chromosomes of the complement (Figure 5c). In the karyotypes of *P. cuvieri* from Araruna (PB), PcP190 chromosomal sites were detected in all the chromosomes at centromeric (chromosome pairs 1, 2, 4 to 11) or centromeric/pericentromeric regions (chromosome pair 3) (Figure 5d). In specimens from Porto Nacional (TO), the PcP190 probe hybridized to the centromeric regions of chromosome pairs 1 through 7, 9 and 10 and to pericentromeric regions of pairs 2, 3, 5 and 7 (Figure 5e).

## Discussion

### Wide distribution of PcP190 satellite DNA

Satellite DNA sequences are present in most eukaryotic organisms, and they are often species-specific [5,10] or present in closely related species [6-9]. In the present study, we detected the presence of PcP190 satellite DNA sequences in all the anurans analyzed, which included six species of *Physalaemus*, but also *L. latrans* and *C. gaudichaudii*. These species are representatives of two families of Anura, i.e., Leptodactylidae and Hylodidae, which are included in the superfamily Hyloidea, together with 17 other families [46]. According to a recent estimation [47], Leptodactylidae and Hylodidae shared a common ancestor approximately 70 million years ago. Therefore, it is likely that the PcP190 satellite DNA has existed since at least that time and remained conserved enough to be recognized in the analyzed species.

Based on the alignment of all the sequences, we can conclude that insertions and deletions are not very frequent in the PcP190 sequences, which is a common characteristic of a number of satellite DNA families [3,48,49]. The sequences of the PcP190 satellite DNA isolated from *P. cuvieri, P. centralis*, *P. albonotatus*, *P. albifrons*, *P. ephippifer, P. marmoratus* and *L. latrans* are conserved in size, which was approximately 190 bp in the vast majority of the sequences. This size is very common in centromeric satellite DNA sequences, for example, the α-satellite DNA of primates [50], the ATOC180 satellite in *Drosophila obscura*, *Drosophila ambigua* and *Drosophila tristis* [51], and the ATCON satellite in *Arabidopsis* [52], among others.

Although there is no universal characteristic among the different types of centromeric satellite DNA, it has been proposed that the size of the satellite DNA monomers are directly related to the required size of a DNA strand to form a centromeric nucleosome [53,54]. This apparent consistency in the size of centromeric satellite DNA sequences may suggest the length required for a proper interaction with specialized histones that form the centromere; in this case, the satellite repetitions would ensure proper separation between these specialized histones during chromatin packaging [54]. In all the *Physalaemus* species analyzed here, the PcP190 satellite DNA was detected in centromeric and pericentromeric regions of constitutive heterochromatin, which was previously detected by C-banding [25,34,35]. Therefore, it is possible that the PcP190 satellite DNA family plays a role in the centromeric chromatin of these anurans, and further studies are necessary to better address this open question.

A distinct region of approximately 74 bps (positions 94–169 in Figure 2) was observed in the sequences, which appeared to be specific to each of the three genera, *Physalaemus*, *Leptodactylus* and *Crossodactylus*. The presence of this divergent block flanked by conserved segments appears to indicate the occurrence of recombination events, which may result in abrupt changes in relatively large segments. However, as the three genera are not closely-related phylogenetically [46], an alternative hypothesis would be that the more conserved regions are under selective pressure, whereas the highly divergent region undergoes frequent point mutations.

A number of studies have identified alternating conserved and variable regions in satellite DNA sequences. In one segment of the MARJA and MPA1 satellite DNA of the *Meloidogyne* nematodes, for example, alternating domains of high and low variability were detected that were conserved both within and between monomer variants in different, but closely-related species [55,56]. Similarly, the 178-bp satellite of *Arabidopsis thaliana* and the human α-satellite also have alternating conserved and variable regions, suggesting that this pattern of non-random variation within the repeating units may be related to the presence of centromeric protein binding sites [57]. Further studies of a larger sample of PcP190 sequences from a wider range of species will be necessary to determine whether the alternating regions present in this satellite DNA do in fact suffer differential selective pressures.

It is also worth noting that species-specific groups were identified in the sequences of three *Physalaemus* species (*P. albifrons*, *P. albonotatus* and *P. marmoratus*), although those of *P. cuvieri, P. centralis* and *P. ephippifer* were distributed in mixed clusters. This result may arise from the fact that different evolutionary rates may have affected the PcP190 family in different species. However, because we could not sample a vast number of sequences, we cannot exclude the possibility that our samples do not represent the full diversity of sequences in the genome of each species.

### Differential amounts of PcP190 sequences in *Physalaemus* species

The results of chromosomal and membrane hybridization experiments were consistent and showed differential amounts of PcP190 sequences in the *Physalaemus* species studied here. The species with lower amounts of PcP190 in their genomes based on the dot-blot analysis were the same as those with fewer chromosomal sites detected by FISH, for example *P. albonotatus*. Likewise, the highest abundance genomic PcP190 sequences were detected by dot-blot in the same specimens in which several chromosomal sites were detected by *in situ* hybridization, for example *P. cuvieri* from Araruna (PB).

Another observation from the FISH experiments was the strong hybridization signal of the PcP190 probe on the pericentromeric region of the short arm of chromosome 3 in *P. cuvieri, P. centralis* and *P. ephippifer.* In the karyotypes of *P. cuvieri* from Palmeiras (BA) [25] and *P. ephippifer* [35], this pericentromeric region on chromosome 3 is the same region that bears the type I 5S rDNA. Because the PcP190 satellite DNA and the 5S gene share 70% similarity [25], we cannot discard the possibility that this strong signal resulted from the cross-hybridization of the PcP190 probe with the 5S rDNA. However, it is not likely, since the hybridization with a type I 5S rDNA probe did not detect any PcP190 satellite regions outside of chromosome 3 [25].

In species that are phylogenetically closely related, deletions and amplifications leading to changes in specific satellite DNA families have been linked to chromosomal evolution [9,22,58]. In the case of the RPCS satellite DNA found in *Ctenomys* species, two evolutionary patterns were detected [9], one of them consisted of a variable number of RPCS copies in species of the same phylogenetic clade with high karyotypic variability, whereas the other pattern, which was also observed in species of the same phylogenetic clade, consisted of a stable number of RPCS copies and stable karyotypes. Based on these data, Slamovits et al. [9] suggested that amplification, deletion and intragenomic rearrangements can promote chromosomal evolution. Conversely, although the amounts of PcP190 satellite DNA varied among the analyzed species of *Physalaemus*, relevant changes in their karyotypes have not been observed in any of them [29,34,35]. However, we cannot rule out the possibility that the differences in the PcP190 clusters in the chromosomes of these *Physalaemus*, as well as the interpopulational differences in *P. cuvieri*, may play or have played an important role in the evolutionary history of these species. An example of such role has already been suggested for *Drosophila*, in which the 359-bp satellite DNA apparently affects or has affected chromosomal segregation in hybrids and, consequently, led to post-zygotic reproductive isolation [59].

Among the *P. cuvieri* populations studied here, only those from Passo Fundo (RS) and Três Lagoas (MS) have not been previously analyzed by classical cytogenetic techniques. Among the other populations, high interpopulational variation in NOR-bearing chromosomes was observed [34]. In individuals from Porto Nacional (TO), for example, in addition to NORs that were dispersed on several chromosomes, a unique C-band pattern was also detected that was mainly characterized by the presence of large pericentromeric blocks on most of the chromosomes [34]. Furthermore, the *P. cuvieri* from Porto Nacional (TO) also has a distinct genetic structure that can be observed using microsatellite markers, suggesting that some of individuals analyzed most likely belong to other species [60].

The majority of the *P. cuvieri* karyotypes analyzed here showed conspicuous differences in the PcP190 hybridization patterns, especially those from individuals from Porto Nacional (TO). The karyotype of these individuals had pericentromeric regions of several chromosomes that hybridized to the PcP190 probe, all of which corresponded to C-bands reported by Quinderé et al. [34]. On the other hand, the same pattern of PcP190 hybridization was seen in the karyotypes of individuals from Uberlândia (MG) and Três Lagoas (MS).

### PcP190 Satellite DNA in the sex chromosomes of *P. ephippifer*

Interesting cytogenetic characteristics of *P. ephippifer* include the heteromorphic sex chromosomes Z and W, which are differentiated mainly by the presence of an additional region containing a NOR and a C-band on the terminal region of the short arm of the W chromosome, despite these chromosomes are similar in size [35]. Additionally, the classical cytogenetic techniques (Giemsa staining, C-banding and the Ag-NOR method) revealed no differences between the long arms of these sex chromosomes, which have a pericentromeric C-band and a terminal NOR [35]. In the present study, however, we detected hybridization of the PcP190 probe to the pericentromeric region of the long arm of the W chromosome that was noticeably stronger than that observed on the long arm of the Z chromosome. The NOR on the long arm of the W chromosome from the female analyzed here was also larger than the NOR on the long arm of the Z chromosome.

Based on these results, we suggest that not only the short arms but also the long arms of the Z and W chromosomes are different. However, the analysis of a larger sample of individuals with several males and females is needed to confirm that the larger PcP190 cluster is exclusively found on the W chromosome.

The accumulation of satellite DNA during sex chromosome differentiation is a common feature of eukaryotes and becomes more apparent when recombination is paused [61,62]. Although this accumulation is intensified on either of the sex chromosomes, it is likely that homologous (or pseudo-homologous) regions contain the same family of satellite DNA, but with different copy numbers and/or arrangements of the clusters (e.g., [63,64]).

## Conclusions

The PcP190 satellite DNA, which was originally found in *P. cuvieri*, was identified in representatives of two families of frogs, Leptodactylidae and Hylodidae, showing that these sequences are widely distributed and have been conserved in these frogs for at least 70 million years. Based on the pattern of non-random variation within the repeating units, we speculate that interplay between stochastic events and selective pressure along of the PcP190 satellite DNA sequences has occurred.

Differences in the chromosomal clusters of the PcP190 satellite DNA are evident among *P. cuvieri* populations and related species, suggesting highly dynamic amplification/deletion events. PcP190 satellite DNA appears to accumulate on the W chromosome of *P. ephippifer*, which may contribute to the differentiation process of the Z and W sex chromosomes in this species.
